# Cellular Response to RGD Peptide Configuration on Gold Nanoparticles: A Surface Chemistry Investigation

**DOI:** 10.1021/acsomega.5c00688

**Published:** 2025-05-14

**Authors:** Melike Sarıçam, Merve Ercan Ayra, Mustafa Culha

**Affiliations:** † Department of Genetics and Bioengineering, Yeditepe University, 34755 Istanbul, Turkey; ‡ 52991 Sabanci University Nanotechnology Research and Application Center (SUNUM), Istanbul 34956, Turkey; § Department of Chemistry and Biochemistry, Augusta University, Augusta, Georgia 30912, United States

## Abstract

Biosystems are exceptional mechanisms for recognizing minute molecular differences in their processes, a principle upon which modern medicine is primarily built. Nanomaterials (NMs), unlike molecules, lack a definite shape and reactivity. Their surface chemical properties serve as the primary element of their behavior in biological environments. Therefore, it is essential to understand how molecular modifications on the surface of NMs influence their functions in biosystems to optimize their use in medical and biomedical applications. Gold nanoparticles (AuNPs) with well-defined surfaces are ideal for systematic surface chemistry studies due to their inertness and low toxicity. In this study, we examined the impact of molecular orientation differences in a peptide with a CRGD sequence and its reverse sequence, DGRC, on the cellular response of A549 (human Caucasian lung carcinoma) and BEAS-2b (human bronchial epithelial cell) cell lines. One end of the peptides contains a cysteine residue to ensure binding to 13 nm AuNP surfaces from that end. When the peptides are conjugated, two distinct surface chemistries are generated: in one case, a surface with one −NH_2_ and one −COOH group creates a neutral charge, while in the other, a surface with two −COOH groups generates a negative charge since the peptides are in the reverse amino acid sequences. We observed that the AuNP-CRGD-NH_2_ conjugate exhibited higher uptake and caused severe cytotoxicity by inducing cell cycle arrest at the G0/G1 phase in A549 cells, whereas no significant harm was detected in BEAS-2b cells compared to the AuNP-CRGD-COOH conjugate. These results strongly suggest that the cellular response to NMs can be effectively modulated through surface chemistry. The AuNP-CRGD-NH_2_ conjugate should be further evaluated for its potential therapeutic effects against lung cancer.

## Graphical abstract

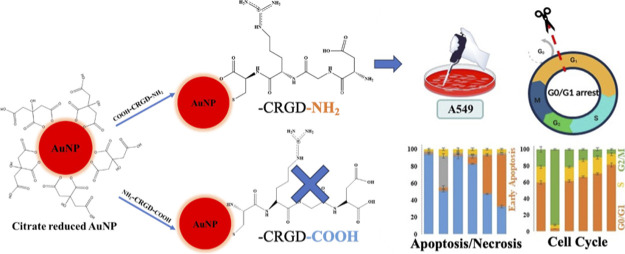

## 1 Introduction

The surface chemistry of nanomaterials (NMs) has been a key area of interest due to its significant impact on their stability, functionality, and toxicity, especially as their applications expand beyond medicine into various other fields.^1–4^ The surface of an NM serves as the interface that dictates its interactions in biological media, offering an opportunity to tailor its functional behavior for targeted applications.^5–9^ Upon introduction into a biological system, NMs undergo a phenomenon wherein a layer of proteins and ionic species, known as the “protein corona,″ forms around them. This protein corona, which strongly depends on the surface chemistry, dramatically influences the stability and functionality of NMs.^10–12^ The dynamic nature of this layer is defined by the degree of adherence of proteins and ionic species to the NM surface. If proteins bind strongly to the surface, a “hard corona” forms, which often disables the surface’s underlying functionality. In contrast, weaker binding results in a “soft corona,″ allowing the surface functionality to remain more accessible due to its dynamic nature. The molecular and ionic composition of the biological medium, along with the surface chemistry of the NM, significantly influences the protein layer’s properties and its subsequent interactions with cells.

Among various NMs, colloidal gold nanoparticles (AuNPs) have been extensively studied due to their ease of synthesis, versatile surface chemistry, biocompatibility, plasmonic properties, and tumor-penetrating ability.^13,14^ Peptide-functionalized AuNPs have also been reported for their applications in cancer diagnosis and therapy.^15–17^ While there is growing interest in understanding the role of surface chemistry in NM toxicity,^18,19^ its influence and modulation on their therapeutic potential remain underexplored.^20,21^ In our earlier studies, we demonstrated how the surface chemistry of colloidal silver nanoparticles (AgNPs) impacts their interactions with living cells.^22^ Furthermore, we demonstrated how the surface chemistry of AgNPs can have a profound effect, even inducing cell death.^23^ More recently, we showed that altering the orientation of hydroxyl (−OH) groups in mono- or disaccharides covalently bound to the surfaces of AuNPs significantly changes the cellular response.^24^

The RGD peptide (arginine, glycine, and aspartic acid) is an adhesion motif found in extracellular matrix (ECM) proteins. This peptide plays a crucial role in cell–cell integration by interacting with integrins.^25,26^ The RGD peptide is a promising targeting molecule for potential therapeutic applications due to its ability to inhibit angiogenesis (through the expression of αvβ3 integrins in most endothelial cancers), tumor growth, and metastasis by binding to αvβ3 integrins.^27^ In one study, it was demonstrated that AuNP-RGD conjugates were taken up five times more efficiently than citrate-capped AuNPs, reaching the cytoplasm and nuclei of cells with a reduced tendency for excretion.^28^ Another study showed that RGD motifs on AuNPs bound to α5- and αv-integrins in MDA-MB-231 cells, resulting in enhanced internalization. In combination with radiation, the presence of AuNP-RGD conjugates decreased cell viability, increased DNA damage, and inhibited the invasive activity of MDA-MB-231 cells.^26^ Additionally, a recent study utilized the RGD motif to target PAD4 inhibitor-loaded spherical and rod-shaped AuNPs to cancer cells, aiming to inhibit tumor growth, prevent lung metastasis, and improve biosafety.^29^ There are several well-documented reviews in the literature discussing RGD peptides and their therapeutic effects.^13,30,31^

In this study, we explored how small molecular alterations at the surface of NMs modulate their functional characteristics. Thus, the RGD peptide and its reverse sequence were conjugated to AuNPs to investigate their potential anticancer effects on A549 (human Caucasian lung carcinoma) and BEAS-2b (human bronchial epithelial) cell lines. The two CRGD peptides, with the same sequence but in reverse amino acid orientations, were custom designed. Both peptides have a cysteine residue at one end to ensure binding to the AuNP surface. When the peptides are conjugated, in one case, the −NH_2_ terminus remains free on the surface, while the −COOH terminus remains free on the surface in the other case. Since aspartic acid remains close to the surface with a free −COOH group, the total surface charge is theoretically neutral for the −NH_2_-terminated surface, while it is negative for the −COOH-terminated surface due to the presence of two −COOH groups. Furthermore, the difference in the bond orientations between the two peptide sequences is expected to result in distinct molecular crowding on the AuNP surfaces. Given the widespread use of RGD peptides for targeting cancer cells, we systematically evaluated the cellular responses to these two chemically distinct AuNP surfaces in terms of cytotoxicity, cellular uptake, and cell cycle effects on the A549 and BEAS-2b cell lines. The results showed that AuNP-CRGD-NH_2_ was highly internalized, caused significant cytotoxicity, and induced cell cycle arrest at the G0/G1 phase in A549 cells, while no notable harm was observed in BEAS-2b cells. These findings highlight the fundamental role of surface chemistry in modulating the therapeutic effects of the NMs. Moreover, the distinct effects of the AuNP-CRGD-NH2 conjugate suggest its potential for further investigation as a therapeutic agent for lung cancer.

## 2 Materials and Methods

### 2.1 Materials

#### 2.1.1 Chemicals

Gold­(III) chloride trihydrate (≥99.9% trace metals basis), methanol (for HPLC, ≥99.9%), hydrochloric acid (36.5–38.0%), sodium hydroxide (BioXtra, ≥98.0%), agarose (powder), Trizma base (powder, ≥99.0%), EDTA (anhydrous, ≥98.5%), acetic acid (anhydrous, ≥99.0%), Triton X-100 (for molecular biology), and ethanol (anhydrous, ≥99.5%) were purchased from Sigma-Aldrich (Darmstadt, Germany). Crystal violet (powder) and trisodium citrate (powder) were purchased from Merck Millipore (Germany). Peptides were synthesized by Alpha DNA (Montreal, BC, Canada) and used as they were received.

#### 2.1.2 Cell Lines

A549 (human Caucasian lung carcinoma) and BEAS-2b (human bronchial epithelial cell) cell lines were purchased from the American Type Culture Collection (ATCC).

#### 2.1.3 Cell Culture Reagents and Kits

Dulbecco’s Modified Eagle Medium (1× DMEM, 4500 mg/L Glucose, l-glutamine, sodium pyruvate, phenol red), Dulbecco’s Modified Eagle Medium/Nutrient Mixture F-12 (DMEM/F-12, 4500 mg/L glucose, l-glutamine, sodium pyruvate, phenol red), fetal bovine serum (FBS), penicillin (10,000 Units/ml), streptomycin (10,000 μg/mL), l-glutamine (200 mM), 1× Trypsin-EDTA (0.25% Trypsin- 0.02% EDTA), and HyClone phosphate-buffered saline (10× PBS) were purchased from Gibco, USA. Colchicine (≥95% HPLC purified, powder) and dimethyl sulfoxide (anhydrous, ≥99.9%) were purchased from Sigma-Aldrich (USA). Tissue culture flasks, well plates, Falcon tubes, serological pipettes, and cryotubes were purchased from TPP, Switzerland. Annexin V-FITC-/PI apoptosis necrosis detection kit was obtained from Calbichem (Germany). Propidium iodide solution (10 mg/mL in water) and ribonuclease A were purchased from Sigma-Aldrich (USA).

### 2.2 Methods

#### 2.2.1 AuNP Synthesis

AuNPs with an average size of 13 nm were synthesized using the Turkevich method.^32^ A solution of 80 mg of gold­(III) chloride trihydrate (HAuCl4·3H2O) in 200 mL of deionized water (dH2O) was heated to boiling with continuous stirring at 1000 rpm using a magnetic stirrer on a hot plate. Once the mixture was boiling, 228.22 mg of sodium citrate dissolved in 20 mL of dH2O was rapidly added to the mixture. The resulting solution was stirred for an additional 15 min. After synthesis, the AuNP suspension was allowed to cool to room temperature and was subsequently characterized.

The concentration of AuNPs in their suspension was calculated using Beer–Lambert’s Law.^33^ First, a series of dilutions of the AuNP suspension (1:2, 1:4, 1:8, and 1:16 v/v) were prepared in dH_2_O to determine the concentration of the AuNPs (*n* = 3). The samples were scanned from 200 to 800 nm in a quartz cuvette by a UV/vis spectrometer. The spectral data of the diluted AuNP suspensions were used for concentration calculations. The number of AuNPs in 1 mL suspension was calculated using a formula reported by Haiss et al., considering the absorbance value of the AuNP suspension at 450 nm.^33^

#### 2.2.2 Surface Modification of AuNPs with CRGD Peptides

The two custom-designed CRGD peptides were used to investigate the cellular response to the chemical differences generated on AuNP surfaces. Table 1 shows the design of the peptides. The RGD has the natural sequence from the −COOH end to −NH_2_; however, the other is in the reverse form from the −NH_2_ end to −COOH. Cysteine (C) at one end is used to establish the Au–S bond via the −SH group.^34^

**Table 1:** Properties of Peptides Used in the Study^a^

code	length (aa)	sequence (from NH_2_ to COOH)	isoelectric point	charge
CRGD-NH_2_	4	DGRC	6.09	0
CRGD-COOH	4	CRGD	6.09	0

a C: cysteine, D: aspartic acid, G: glycine, R: arginine.

The lyophilized peptides were dissolved in dH_2_O based on the manufacturer’s instructions. First, a stability study of AuNP colloidal suspension at varying pHs by adding 0.05 M HCl or NaOH was performed to find out the pH range in which the colloidal suspension was stable. It was found that the suspension was stable in the range of 3.0–11.8. Then, the peptide solutions (1 mg/mL, 20–25 μL) (3 trials from each peptide, *n* = 3) with increasing concentrations were added into AuNP suspensions (10 nM, 1 mL) and were shaken overnight. The most stable pH range for CRGD-NH_2_ was found to be 11.0–11.8, and for CRGD-COOH, the original AuNP suspension pH of 5.9 was used and found to be stable as no aggregation was observed in the range of 5.5–11.8.

##### 2.2.2.1 Characterization of AuNPs and Their Peptide Conjugates

The colloidal AuNPs and their peptide conjugates were characterized by TEM, UV/vis spectroscopy, dynamic light scattering (DLS), agarose gel electrophoresis, and surface-enhanced Raman scattering (SERS). For SEM, a few microliters of AuNP suspension were spotted onto TEM grids, and the images were acquired at varying magnifications by using a JEOL JEM 100C TEM instrument. For UV/vis spectroscopic characterization, a Lambda 25, PerkinElmer, Foster City, CA, USA, spectrometer was used. A 1:10 diluted AuNP suspension in dH_2_O was used by scanning in the range of 200–800 nm in a 1 cm path length quartz cuvette. For size and surface charge determination of AuNPs in suspension, a DLS instrument (ZetaSizer Nano ZS, Malvern Instruments, Malvern, UK, equipped with a 4 mW He–Ne laser with a 173° scattering angle) was used. Again, a 1:10 dilution of AuNP suspension was used for measurements in polystyrene cuvettes at ambient conditions.

The binding efficiency and density of peptides on AuNPs were monitored using 1% Agarose gel electrophoresis. The naked AuNP and AuNP–peptide conjugate suspensions were centrifuged at 13000 rpm for 20 min. The supernatants were discarded, and the pellets were dissolved in 1 mL of dH_2_O. The samples were centrifuged two more times, and 0.98 mL of supernatant was carefully removed. The pellets were resuspended in the remaining 20 μL of supernatant by soft vortex shaking. An 80 mg agarose powder was melted in 80 mL of 1× TAE buffer solution (40 mM Tris-acetate and 1 mM EDTA) by microwaving. Since AuNP suspension has a reddish/purple color, no ethidium bromide (EtBr) was added to the gel. The gel cassette was placed in an electrophoresis chamber, and 1× TAE buffer solution as the running buffer was dropped into the chamber to cover up the gel. The chamber was fitted with Pt electrodes of a DC supply (PowerPac Basic Power Supply, BioRad). The gel was run at 100 V for 1.5 h.

Since AuNPs are SERS active, the spectra obtained from the AuNP–peptide conjugates can be used to investigate the peptide binding onto AuNPs. A Renishaw inVia Reflex Raman spectrometer equipped with a high-speed encoded Streamline stage and Leica DM2500 upright microscope, UK, was used for the measurements. For both the naked AuNPs and the AuNP–peptide conjugates, the suspensions were centrifuged at 13,000 rpm for 20 min, and the pellets were redispersed in 1 mL of d. i. water and washed two times by repeating the centrifugation and resuspension. From the suspensions, 2 μL was dropped on CaF_2_ slides, and at least ten separate areas of 10 × 10 μm^2^ with a laser spot size of 1.5 μm were mapped under a 50× objective using an 830 nm photodiode laser with 4 s of laser exposure and 150 mV laser power. The acquired spectra were preprocessed through baseline subtraction, cosmic-ray removal, and smoothing. Then, the spectra obtained from the mapped areas were averaged and normalized.

#### 2.2.3 Cell Culture Studies

The details of cell culture studies, cellular uptake, apoptosis/necrosis assay, clonogenic assay, and cell cycle evaluation are provided in the Supporting Material.

## 3 Results and Discussion

### 3.1 Surface Characterization of AuNP-CRGD Conjugates

A representative TEM image of AuNPs, along with UV/vis and DLS spectra of their colloidal suspension, is shown in Figure 1. The TEM image reveals an average particle size of 13 nm, while the UV/vis and DLS spectra exhibit characteristics typical of a colloidal AuNP suspension synthesized via the citrate reduction method.

**Figure 1:**
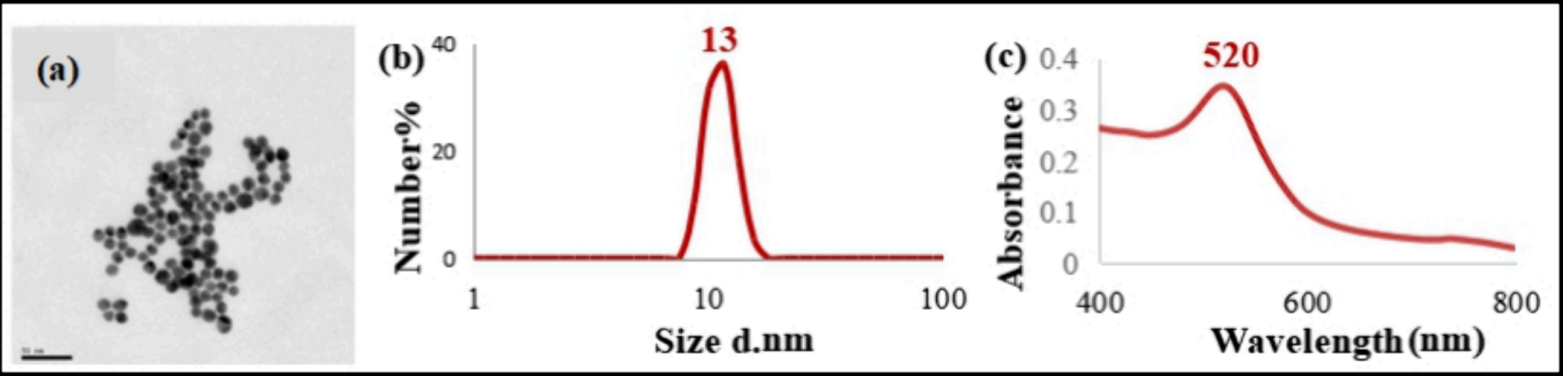
(a) TEM image of AuNPs and (b) DLS and (c) UV/vis spectra of the AuNP suspension.

As key parameters in this study, the concentration and number of AuNPs in the suspension were determined using Beer–Lambert’s Law, yielding values of 10 nM and 5.37 × 10^12^ particles, respectively. Detailed concentration calculations are provided in the Supporting Information (see Figure S1). The concentrations of AuNP–peptide conjugates were assumed to be equivalent to those of the original 13 nm AuNP suspension. This assumption was based on minimal AuNP loss during the washing step, where extra caution was taken to mitigate losses. Any minor loss of AuNPs was deemed negligible, given the experimental uncertainty. Note that aggregation during pellet redispersion could impact AuNP number and concentration; however, precautions were also taken during these steps to minimize such effects.

The conjugation of peptides to AuNPs is illustrated in Figure 2. While the RGD peptide sequence remains unchanged, the bond orientations and charges at the free ends differ. The conjugation of these two peptides creates a chemically distinct environment on the AuNP surfaces, including the charge. A cysteine residue was added to the end, expected to bind to the AuNP surface to ensure proper attachment. In one configuration, the surface charge is primarily neutral due to the presence of one −NH_2_ and one −COOH group, whereas, in the other, the charge is predominantly negative due to the two −COOH groups at neutral pH.

**Figure 2:**
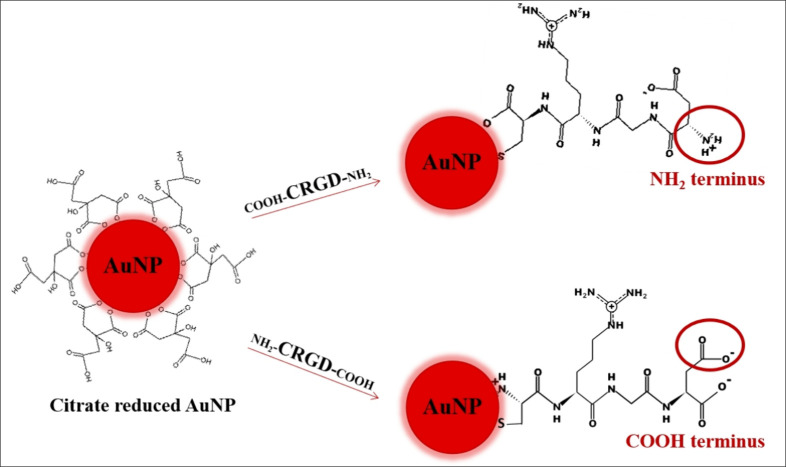
Representation of CRGD peptide conjugation on the AuNP surfaces. C: Cysteine, D: aspartic acid, G: glycine, R: arginine.

The pH of the AuNP colloidal suspension and the pI of a peptide or protein play crucial roles in their conjugation onto AuNPs. It has been reported that the pH of a suspension close to the pI enhances the interaction and, consequently, effective binding.^35^ The pI of the CRGD peptides used in this study was 6.09, while the pH of the synthesized AuNP suspension was measured at 5.9. Therefore, it was expected that the AuNPs would conjugate with CRGD peptides in their original suspension. However, aggregation occurred immediately after the addition of the CRGD-NH_2_ solution, whereas no aggregation was observed with the addition of the CRGD-COOH solution (see Figure S2). At pH 5.9, the suspension is slightly acidic. When CRGD-NH_2_, carrying two −NH_2_ groups, is added, these amino groups are predominantly protonated and positively charged. As a result, the AuNPs aggregate quickly, leaving insufficient time for the −SH group of cysteine to interact with the AuNP surfaces effectively. Therefore, a pH optimization study was conducted to identify the most suitable pH for peptide conjugation. Initially, a qualitative assessment was performed by observing color changes in the AuNP suspensions upon peptide addition. Figures S3, S4, and S5 show white-light images of the suspensions following the peptide introduction. The pH at which no aggregation occurred was considered optimal for conjugation, and further quantitative analyses were conducted (see the Supporting Information for details). It was determined that the ideal pH values for conjugation with CRGD-NH_2_ and CRGD-COOH were 11.0 and 5.9, respectively. The results indicate that the adsorption of −SH groups is influenced not only by the pH but also by the presence of −NH_2_ or −COOH groups on the cysteine side. The adsorption of −SH groups on AuNP surfaces is pH-dependent.^36^ At lower pH levels, it is primarily coordinate-like, whereas at higher pH levels, the bond becomes more covalent in nature, resulting in stronger attachment. In the case of CRGD-NH_2_, at pH 11.0, the −COOH (C terminus) group on the cysteine side is mostly ionized and negatively charged (see Figure 2), further facilitating the peptide adsorption.^37^ In the case of CRGD-COOH, at pH 5.9, the −NH_2_ (N terminus) group on the cysteine side is predominantly positively charged, enhancing peptide adsorption as the positively charged amino group is easily adsorbed through electrostatic interaction with the gold surface.^38^ The guanidinium group of arginine can also play a role in binding to AuNP surfaces, depending on the pH of the suspension, as it is the second amino acid from the binding side. However, its distance and the molecular crowding on the surface may decrease its influence. It was found that 20 μL of 1 mg/mL was enough to cover the surfaces of 1 mL of AuNP suspension containing 10 nM (5.37 × 10^12^ particles). The details of this optimization are provided in the Supporting Material.

Figure 3 shows the further characterization data for the peptide-conjugated AuNPs with UV/vis spectroscopy, DLS, 1% Agarose gel electrophoresis, and SERS. The characteristic SPR bands of the suspensions of naked AuNPs, AuNP-CRGD-NH_2_, and AuNP-CRGD-COOH conjugates are observed at 519, 520, and 522 nm, respectively, as seen Figure 3a. A red shift from 1 to 3 nm in the SPR bands indicates the conjugation. The DLS data in Figure 3b shows that the AuNP-CRGD conjugates have larger sizes than the naked AuNPs. The stability of the conjugate’s suspensions was studied at around the visually observed pH values to ensure their stability. Further DLS data can be found in the Supporting Information and in Table S1. To investigate the CRGD peptide density on each AuNP in the suspension, the AuNPs were loaded on 1% Agarose gel electrophoresis, and their white light image is given in Figure 3c. The naked AuNPs precipitated in the well because of the high salt content in the TAE buffer. On the other hand, AuNP–peptide conjugates run through the gel as a narrow and dense band, indicating that nearly all AuNPs in the suspension had very similar surface charges and accordingly suggesting about the same number of CRGD peptides on the AuNP surfaces. Besides, the AuNP-CRGD-NH_2_ conjugate travels slower than the AuNP-CRGD-COOH conjugate through the gel, indicating their more positive surface charges after they are loaded into the gel. The slower movement of the AuNP-CRGD-NH_2_ conjugate can be attributed to the removal of the negatively charged citrate ions during the washing step and their release after loading into the gel since they are electrostatically bound to the AuNP-CRGD-NH_2_ conjugate surface. Although it was not possible to calculate the peptide density on AuNPs, the gel electrophoresis results show that the peptide surface coverage is good. Finally, SERS was used to investigate the binding of peptides to AuNPs. Figure 3d shows the comparison spectra of naked and peptide-modified AuNPs. SERS is a very sensitive technique to acquire very valuable molecular information from the molecular structures on the surface of noble metal NPs, such as AuNPs. However, the SERS activity of AuNPs, aggregation status, interaction degree, density, and orientation of molecules on the surface can have a dramatic effect on the spectrum. Besides, since the SERS spectral acquisition was made from the dried droplets of the colloidal suspensions, they may not represent the molecular status in solution. For example, the band observed in the range of 500–535 cm^–1^ and attributed to −S–S– vibrations is highly possible that it was formed during drying of the droplets on the CaF_2_ surface as a result of oxidation of the cysteine residue. The other important observation is that the intensity difference between AuNP-CRGD-COOH and AuNP-CRGD-NH_2_, which is attributed to the aggregation status of the AuNPs during droplet drying as a result of the surface charge difference. The following band assignment is based on the spontaneous RS band assignments.^39^ Although a direct comparison of RS and SERS band assignments cannot be made and a shift to lower wavenumbers in SERS measurements is observed as the bonds are either in touch or very close to the metal surface, the presence of the bands around the expected wavenumbers can provide valuable information about the status of the molecules on the surface. The bands at 638–650 cm^–1^ are attributed to C–S gauche vibrations, at 713–745 and 817–840 cm^–1^ to C–N stretching and deformative vibration of amine groups, respectively, at 853–1002 cm^–1^ to C–C stretching, at 1160–1185 cm^–1^ to C–N stretching, at 1221–1322 cm^–1^ to amide III vibrations, and at 1331 cm^–1^ to C–H vibrations. The evaluation of the SERS data supports the presence of the peptides on AuNPs. Overall, all data strongly support the binding of peptides to the AuNP surfaces, and one can safely make the assumption that their orientations are also as expected.

**Figure 3:**
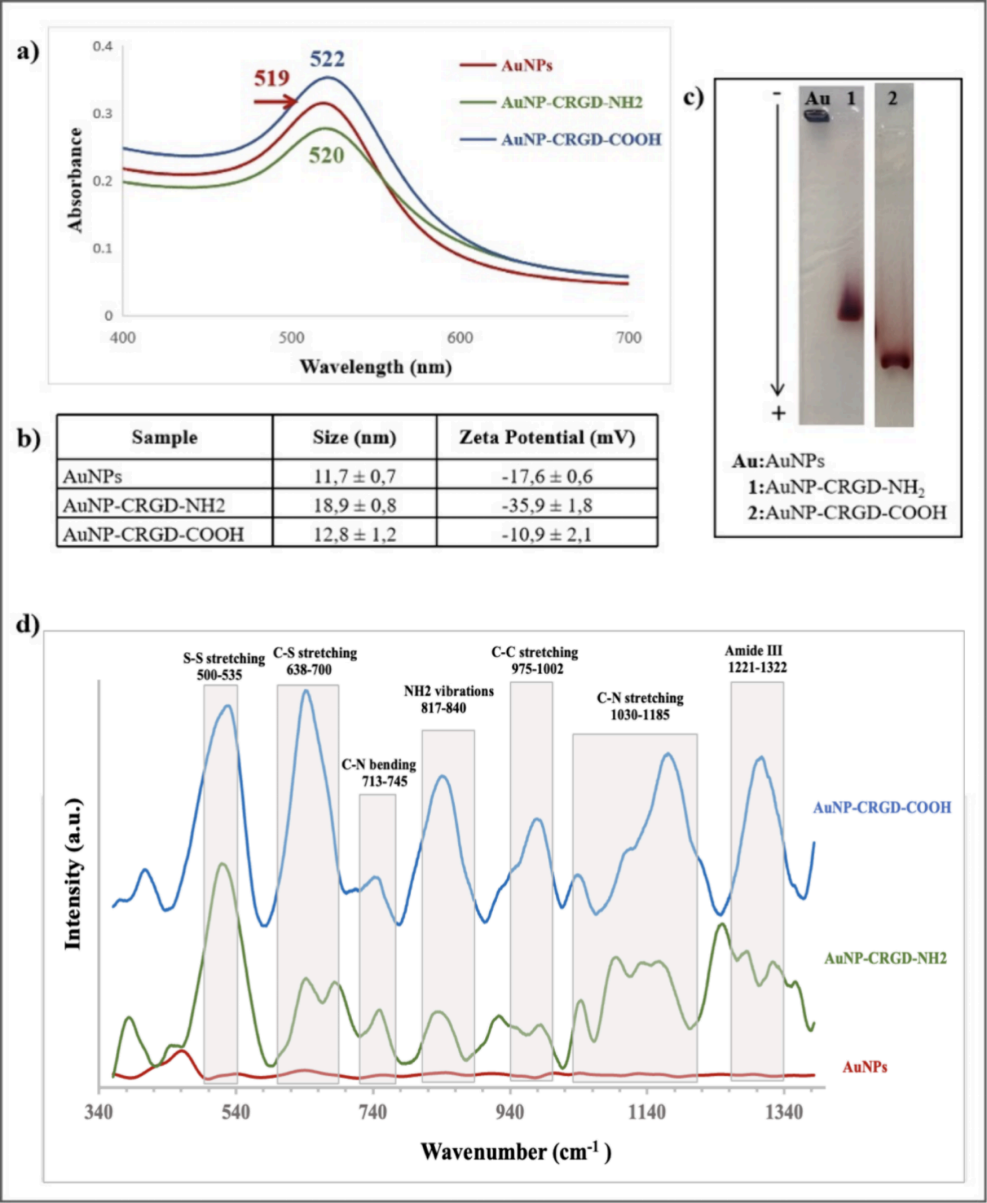
Characterization data for AuNP–peptide conjugates. (a) UV/vis spectra, (b) DSL hydrodynamic size averages and zeta potentials, (c) white light image of Agarose gel, and (d) SERS spectra.

### 3.2 Cellular Uptake Studies

As seen in Figure 3b, AuNP-CRGD-NH_2_ has a more negative surface charge as compared to the AuNP-CRGD-COOH case. “The surface charge of the conjugates is critically important, influencing the protein corona formation. A soft protein corona formation in both cases is expected, perhaps with a softer one on AuNP-CRGD-NH2, as it has a more negative surface charge in its stable suspension before adding into cell culture medium.^40^ The surface charge and size of the AuNPs and peptide conjugates in two different cell culture media are listed in Table S2. When the concentration of the conjugates in DMEM + 5% FBS was varied, a size increase with the AuNP-CRGD-NH_2_ and a size decrease with the AuNP-CRGD-COOH were observed. However, this trend tends to be opposite in the case of DMEM/F12 + 10% FBS. The surface charge changes do not fluctuate significantly in all cases. Thus, the internalization of both conjugates can be considered comparable from a surface charge point of view. The cellular uptake of AuNPs was analyzed by flow cytometry since the increase in cell granulation caused by NPs internalized into the cells or attached to the cell membrane can be detected by an increase in the side-scattered light (SSC) intensity registered from the flow cytometry technique. SSC intensity graphs of A549 and BEAS-2b cells exposed to 0.1, 0.5, 1.0, and 2.5 nM naked AuNPs, AuNP-CRGD-NH_2_, and AuNP-CRGD-COOH conjugates are given in Figure 4. A549 and BEAS-2b cells showed 6 and 8% granulation in negative conditions, respectively. After 24h treatment, A549 cells significantly internalized all concentrations of naked AuNPs and AuNP-CRGD-NH_2_ and high concentrations of AuNP-CRGD-COOH. On the other hand, BEAS-2b cells internalized both AuNP-CRGD conjugates significantly in comparison to naked AuNPs. A higher cellular uptake by BEAS-2b cells was observed with 2.5 nM AuNP-CRGD-COOH. As seen, while A549 cells internalize the AuNP-CRGD-NH_2_ conjugate more, BEAS-2b cells internalize the AuNP-CRGD-COOH conjugate more.

**Figure 4:**
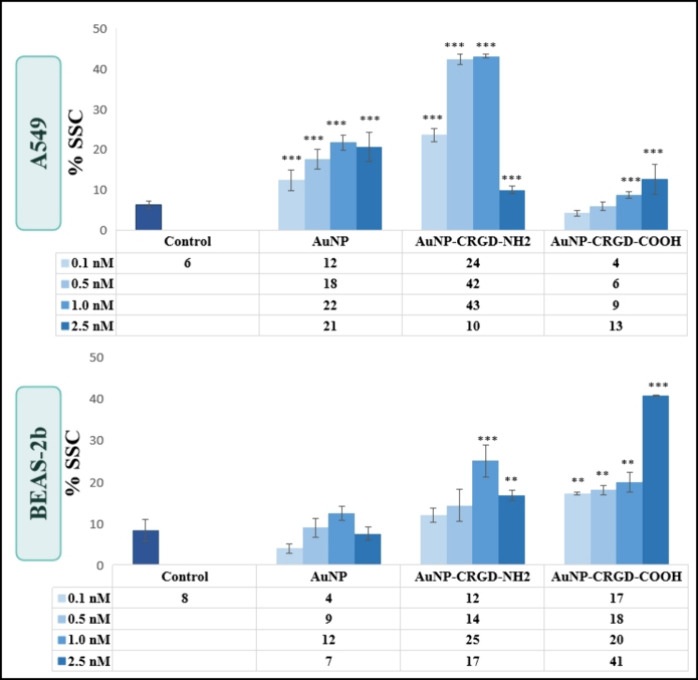
SSC intensity graphs of A549 and BEAS-2b cells exposed to 0.1, 0.5, 1.0, and 2.5 nM naked AuNPs, AuNP-CRGD-NH_2_, and AuNP-CRGD-COOH. Statistically significant changes compared to negative control cells were calculated by two-paired Student’s *t* test, and marked with stars, * for *p* ≤ 0.05, ** for *p* ≤ 0.01, and *** for *p* ≤ 0.001.

### 3.3 Apoptosis/Necrosis Assay

In order to determine the cytotoxicity and the death profiles of the cells upon exposure to AuNP–peptide conjugates, an apoptosis/necrosis assay was performed, and the results are given in Figure 5. 10% DMSO was utilized as a positive control and induced A549 cells to early apoptosis and BEAS-2b cells to late apoptosis. The cell viability of A549 cells was affected by both naked AuNPs and AuNP-CRGD-NH_2_ conjugate. The naked AuNPs created cytotoxicity in A549 cells in parallel to a decrease in their exposed concentration to cells as the cell viability reduced up to 50% after incubation with 1.0 nM. On the other hand, AuNP-CRGD-NH_2_ conjugates caused cytotoxicity in A549 cells with the increased concentration as the live cells decreased to 30% after the treatment with a 2.5 nM dose. Besides, AuNP-CRGD-COOH conjugates caused no cytotoxicity in A549 cells. By considering BEAS-2b cells, neither naked AuNPs nor AuNP–peptide conjugates led to significant cytotoxicity. Consequently, AuNP-CRGD-NH_2_ conjugates created significant cytotoxicity in A549 cells, whereas they did not affect the viability of BEAS-2b cells.

**Figure 5:**
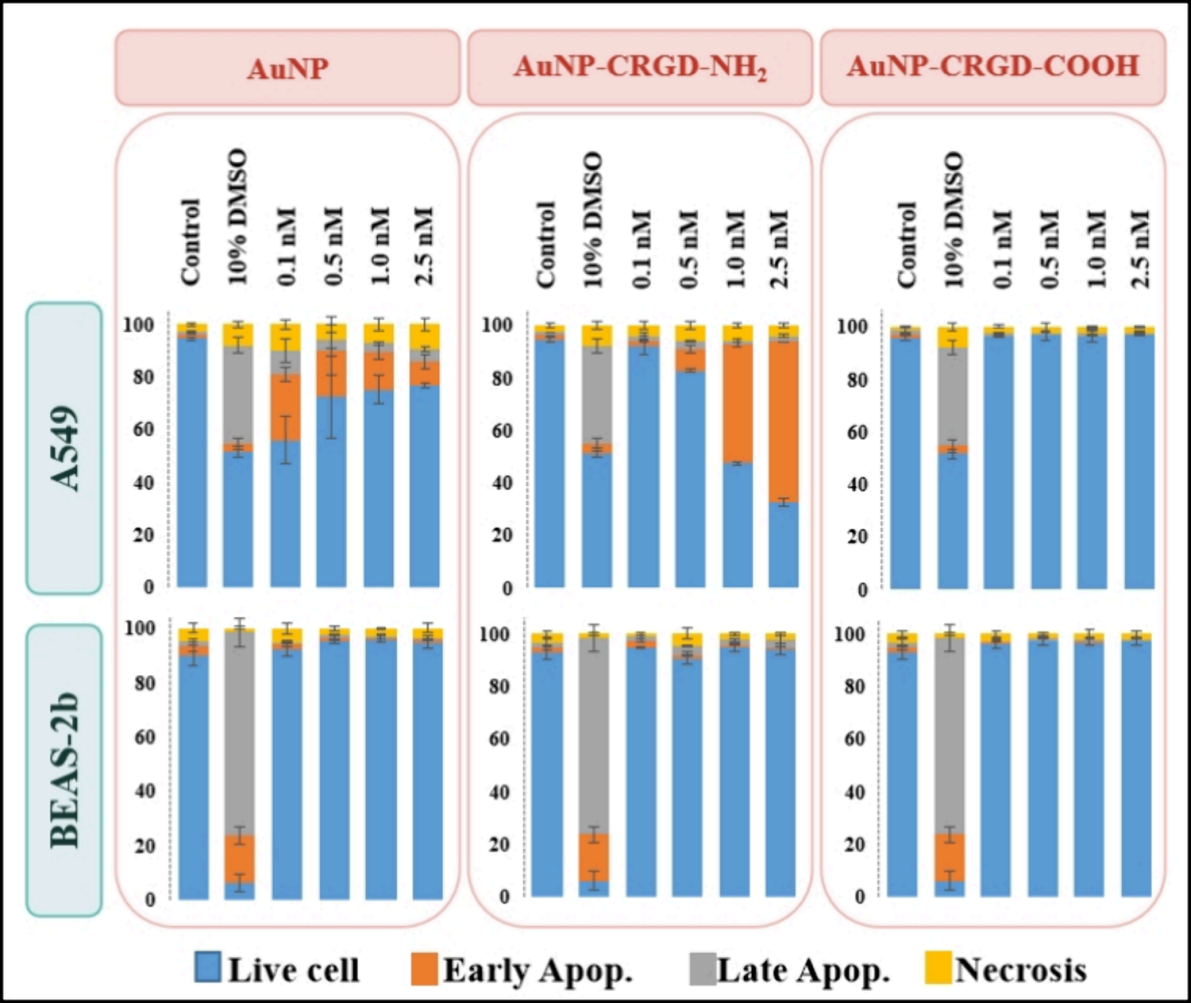
Apoptosis/necrosis assay results for A549 and BEAS-2b cells treated with 0.1, 0.5, 1.0, and 2.5 nM naked AuNPs, AuNP-CRGD-NH_2_, and AuNP-CRGD-COOH conjugates. Positive control was 10% DMSO.

### 3.4 Clonogenic Assay

The cell survival profile upon exposure to NPs is an important phenotypic measurement to obtain information on whether the NPs induced or prevented toxicity.^41^ It was expected that the colony formation ability or forming colonies should decrease in parallel with increasing the concentration of NPs. Thus, with the clonogenic cell survival assay, the long-term cytotoxicity of NPs can be evaluated. Therefore, the colony formation ability of A549 and BEAS-2b cells exposed to 0.1, 0.5, 1.0, and 2.5 nM AuNPs was investigated, and the assay results are provided in Figure 6. 10% DMSO was utilized as a positive control, and the presence of DMSO in the medium resulted in no colony formation by all cell types. After treatment with naked AuNPs, the colony number of A549 cells decreased. However, they were still considered as clonogenic since they underwent more than 2–3 mitoses. When the effect of the AuNP-CRGD conjugates is compared, while the A549 cells cannot survive with the AuNP-CRGD-NH_2_ exposure, the cells exposed to AuNP-CRGD-COOH created more colonies than the negative control. BEAS-2b cells exposed to naked AuNPs could form significant colonies at a concentration of only 0.1 nM. BEAS-2b cells treated with AuNP-CRGD-NH_2_ up to 1.0 nM can be considered as clonogenic since the single BEAS-2b cells generated colonies to survive in the mentioned condition. Nevertheless, BEAS-2b cells were found to be unable to divide after their exposure to AuNP-CRGD-COOH. As a result, no colony formation ability of A549 cells exposed to AuNP-CRGD-NH_2_ was observed, while BEAS-2b cells could survive under the same conditions.

**Figure 6:**
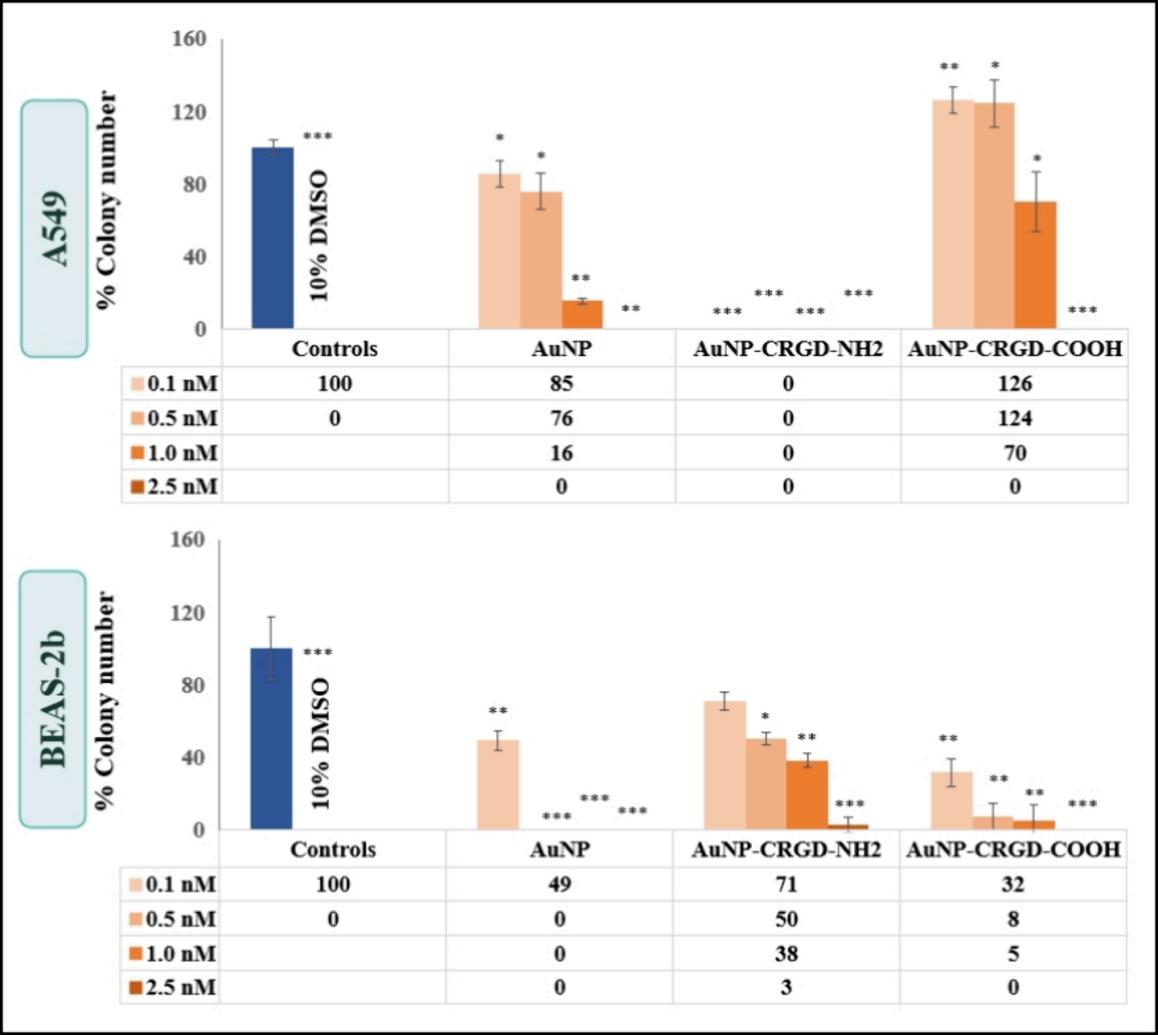
Clonogenic assay results for A549 and BEAS-2b cells treated with 0.1, 0.5, 1.0, and 2.5 nM naked AuNPs, AuNP-CRGD-NH_2_, and AuNP-CRGD-COOH. Positive control was 10% DMSO. Statistically significant changes compared to negative control cells were calculated by two-paired Student’s *t*-test, and marked with stars, * for *p* ≤ 0.05, ** for *p* ≤ 0.01, and *** for *p* ≤ 0.001.

### 3.5 Cell Cycle Progression

The cell cycle progression of A549 and BEAS-2b cells exposed to 0.1, 0.5, 1.0, and 2.5 nM naked AuNPs, AuNP-CRGD-NH_2_, and AuNP-CRGD-COOH was studied, and the results are provided in Figure 7. Colchicine blocking at a concentration of 0.1 μM at the G2/M phase was used as a positive control, and it arrested 85–90% of all cell types at this phase. As seen, the cycle of A549 cells was significantly affected by naked AuNPs and AuNP-CRGD-NH_2_. The higher doses of naked AuNPs led to more than 10% of A549 cells to arrest at the G0/G1 phase. However, the most arrest in the A549 cell cycle was observed after the treatment with a 0.5 nM AuNP-CRGD-NH_2_ conjugate, as approximately 20% of A549 cells treated with its 2.5 nM dose was blocked at the G0/G1 phase. No significant change with A549 cells was observed in the case of AuNP-CRGD-COOH. The cycle of BEAS-2b cells was influenced by only AuNP–peptide conjugates. Concentrations higher than 0.5 nM AuNP conjugates created a severe cell cycle arrest at the G0/G1 phase in BEAS-2b cells. The findings demonstrate that the AuNP-CRGD-NH_2_ conjugates are effective against lung cancer cells up to 0.5 nM, and their potential as therapeutic agents should be further investigated.

**Figure 7:**
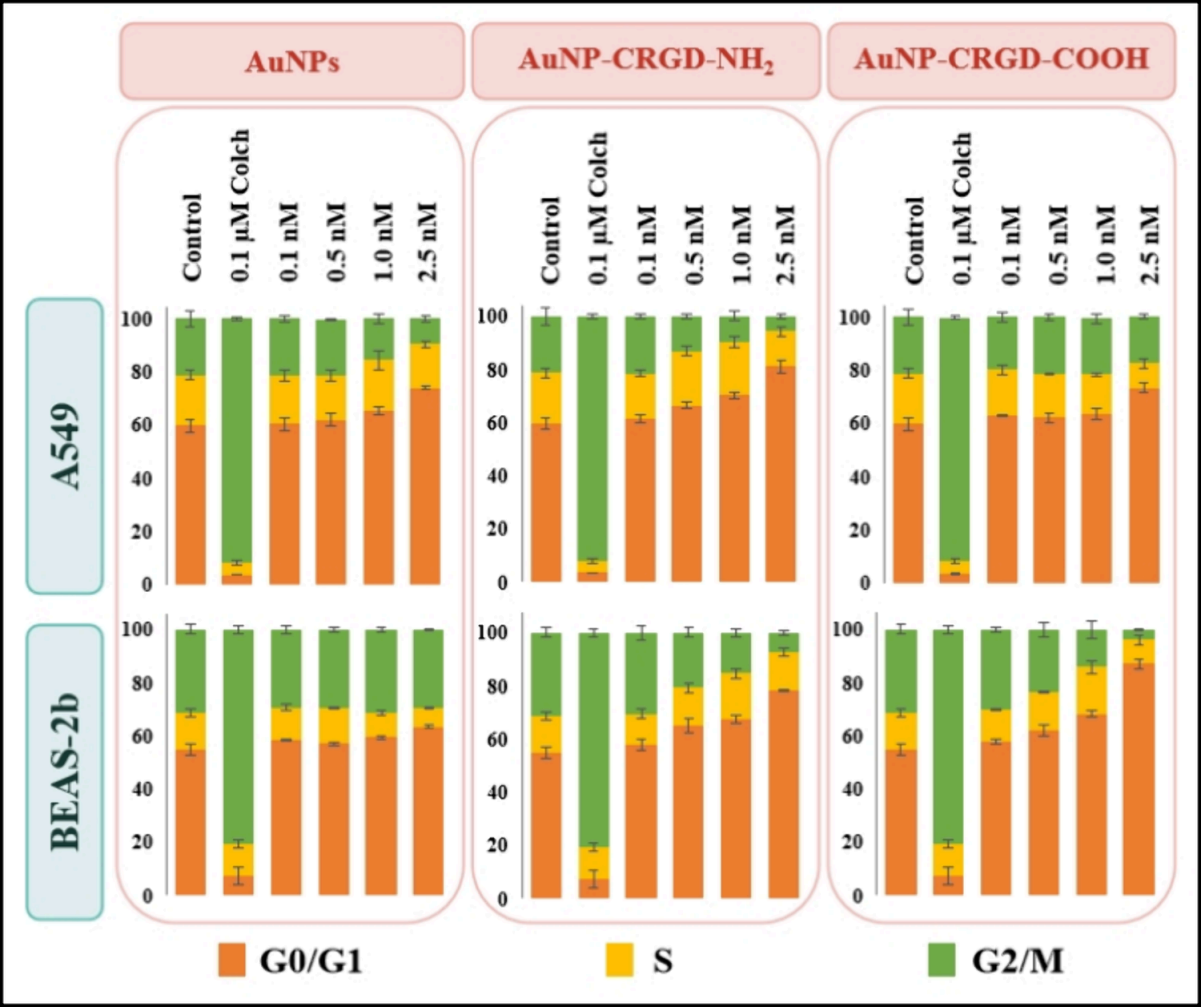
Cell cycle progression of A549 and BEAS-2b cells treated with 0.1, 0.5, 1.0, and 2.5 nM naked AuNPs, AuNP-CRGD-NH_2_, and AuNP-CRGD-COOH. Positive control was 0.1 μM colchicine.

## 4 Conclusions

In this study, we demonstrated how the surface chemistry of an NM influenced its function using two separate designs of the RGD peptide, which are different not only in generated surface charge but also in molecular orientation adjacent to the charged groups protruding to the surface. Even though both AuNP-CRGD conjugates did not cause any cytotoxicity on BEAS-2b cells, the AuNP-CRGD-NH_2_ conjugate was highly uptaken and created severe cytotoxicity and G0/G1 phase cell cycle arrest on A549 cells. This study suggests that the surface chemistry of NPs can be used to modulate their action for the purpose for which they are aimed for. In conclusion, this study found that AuNP-CRGD-NH_2_ conjugates showed a significant therapeutic effect on cancer cells as a result of changes in the surface chemistry of AuNPs. Thus, the AuNP-CRGD-NH_2_ conjugate should be further investigated for lung cancer therapy, considering the fact that the World Health Organization (WHO) declared lung cancer patients were the greatest risk group during the COVID-19 pandemic and called for new effective therapies. Our future studies will continue to evaluate the performance of this conjugate as a therapeutic agent.

## Supplementary Material



## Notes

Calculation of AuNP suspension concentration (Figures S1 and S2); conjugation pH determination (Figures S3–S5); optimization of AuNP conjugation with CRGD peptides and their characterization (Figures S6–S8, Tables S1 and S2); and cell culture experimental procedures for cellular uptake, apoptosis/necrosis assay, clonogenic assay, and cell cycle evaluation (PDF)
