# Supplementary material for: Cellular Response to RGD Peptide Configuration on Gold Nanoparticles: A Surface Chemistry Investigation
Source: ACS Omega. 2025 May 14;10(20):20487–96. doi: 10.1021/acsomega.5c00688 (PMC12120608; doi:10.1021/acsomega.5c00688)
Supplement: Supplementary file 1 [file ao5c00688_si_001.pdf]

SUPPLEMENTARY MATERIALS

Cellular response to RGD peptide configuration on gold nanoparticle: A surface chemistry investigation

Melike Sarıçam[1], Merve Ercan Ayra[1], Mustafa Culha[2,3*]

[1] Department of Genetics and Bioengineering, Yeditepe University, 34755, Istanbul, Turkey
[2]Sabanci University Nanotechnology Research and Application Center (SUNUM), Istanbul 34956, Turkey
[3]Department of Chemistry & Biochemistry, Augusta University, Augusta, GA, 30912, USA

*mculha@augusta.edu/mustafa.culha@sabanciuniv.edu

Calculation of AuNP Suspension Concentration

The size and concentration are the key parameters of AuNPs in their applications. Since the AuNPs can be synthesized in a wide range of sizes by several synthesis protocols, their concentration determination becomes troublesome. Thus, the concentration of synthesized spherical AuNPs in suspension is determined by Beer-Lambert's Law. In this approach, the concentration of AuNPs in suspension can be estimated by measuring the SPR value of AuNPs by UV/Vis spectroscopy [1]. Therefore, the diluted AuNP suspensions in water were prepared as 1:2, 1:4, 1:8 and 1:16 in triplicates. Then, their absorbance values at SPR peak, 519 nm, were recorded three times. The obtained nine measurements for each dilution factors were averaged. Beer-Lambert's law is referred as:

$$A = C \times \varepsilon \times l$$

where A is absorbance of AuNPs suspension, C is the concentration of AuNPs in suspension, ε is extinction coefficient of AuNPs and l is the path length of cuvette, which was 1 cm. The slope of absorbance vs dilution factor graph of AuNPs was calculated as 3.49, and this slope was equal to ε of AuNPs in suspension from formula ε=A/DF. By referring ε values from literature, ε of around 13 nm sized AuNPs has 10^8 M^{-1} cm^{-1} unit (1). Moreover, A_{spr}/A_{450} ratios discussed by Haiss et al. to determine the concentration of 12 and 14 nm AuNP suspension were 1.56 and 1.61 respectively. The ratio of synthesized 13 nm AuNPs was calculated as 1.57, which is consistent with the literature values. Therefore, ε was assessed as 3.49×10^8 M^{-1} cm^{-1}. After the determination of ε, the concentration of AuNP suspension was calculated with Beer-Lambert's Law. The absorbance values at SPR peak and ε of 13 nm AuNPs were placed in the formula and the concentration of synthesized 13 nm AuNPs suspension was found as 10 nM.

In the next step, the AuNP numbers in 1 ml suspension was determined by using a formula, suggested by Haiss et al., as shown in Figure S1. Based on this formula, absorbance value

at 450 nm was crucial to designate the AuNP numbers in suspension. As a result, 5.37×10^{12} 13 nm sized AuNPs were suspended in 1 ml suspension.

The number of AuNPs in 1 ml colloidal suspension:

$$N=\frac{A_{450}\times10^{14}}{d^2[-0.295+1.36\exp(-(\frac{d-96.8}{78.2})^2)]}$$

$N=5.37\times10^{12}$ **AuNPs in 1 ml suspension**

Figure S1. Determination of AuNP numbers in 1 ml suspension. A_{450} was 1.1026 of 1:10 diluted AuNP suspension and d was equal to 6.5 nm.

Investigation of effect of Cysteine location at peptide side to the conjugation

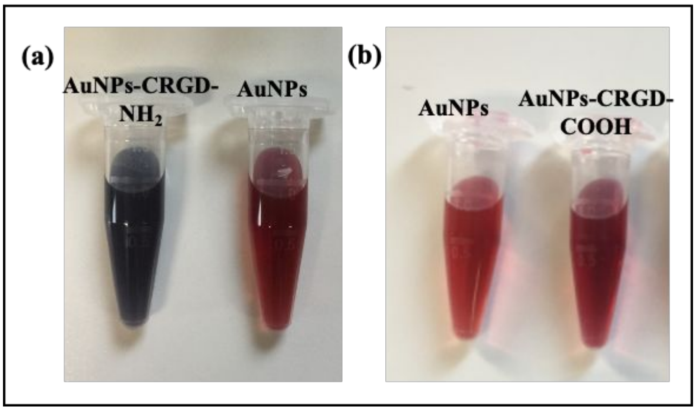

Figure S2. White light images of AuNPs suspensions after addition of **a)** CRGD-NH_2 and **b)** CRGD-COOH.

Conjugation pH Determination

In order to find the optimum pH for AuNP-Peptide conjugation, the pH of AuNP suspension was adjusted before addition of CRGD peptides. Firstly, the stability of bare 13 nm sized AuNPs at different pHs was determined. The pH of original AuNP suspension was measured as 5.9. By adding 0.1 NaOH or 0.1 HCl dropwise, the pH of AuNP suspensions was measured by pH meter. Between pH 3.0 and 11.8, the AuNP suspension was clear, AuNPs in suspension was stable and no AuNPs aggregated, as seen in Figure S2.

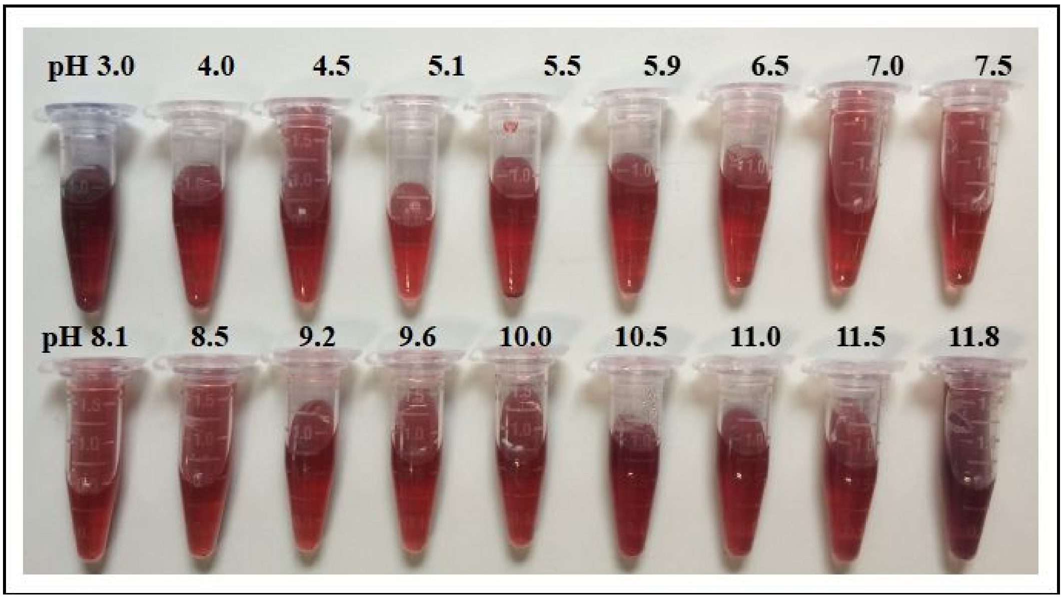

Figure S3. AuNP suspensions at several pHs.

A 20 µl of 1 mg/ml CRGD-NH_2 was added into 1 ml of 10 nM 13 nm AuNP suspensions, whose pHs were in the range of 3.0 to 11.8. After the overnight shaking, the suspension colors changed as seen in Figure S3. A precipitation at pHs lower than 10.0 was observed indicating that AuNPs were possibly functionalized with the CRGD-NH_2 at pHs higher than 10.0.

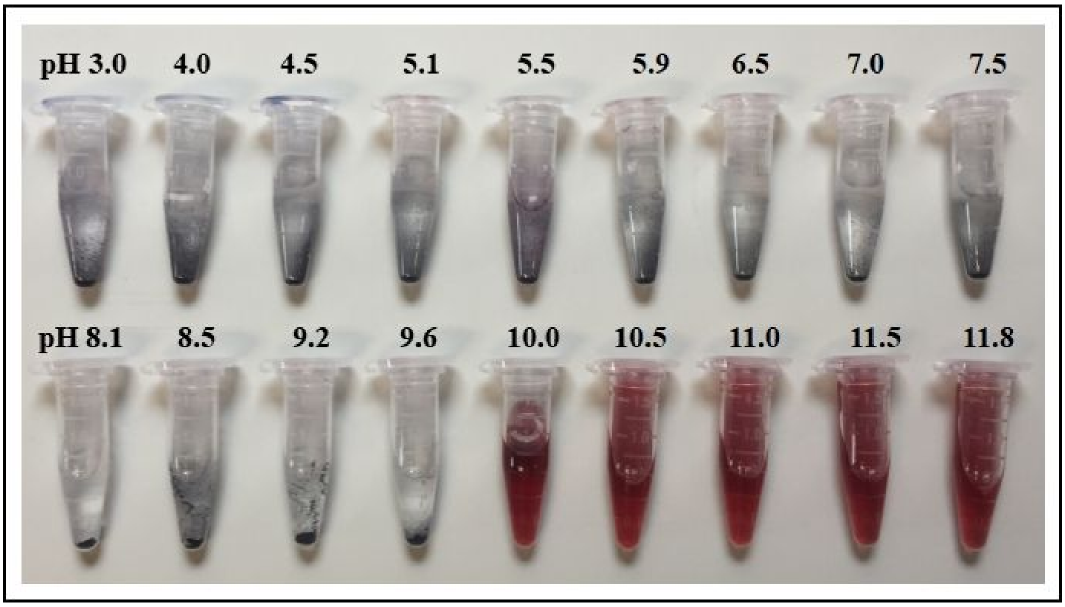

Figure S4. AuNP suspension color change after addition of CRGD-NH_2 solution.

A 20 µl of 1 mg/ml CRGD-COOH was added into 1 ml of 10 nM 13 nm AuNP suspensions, whose pHs were from 3.0 to 11.8. After overnight shake, the suspension colors changed as

seen in Figure S4. The suspension was stable at pHs higher than 5.5 indicating possible conjugation of AuNPs with CRGD-COOH.

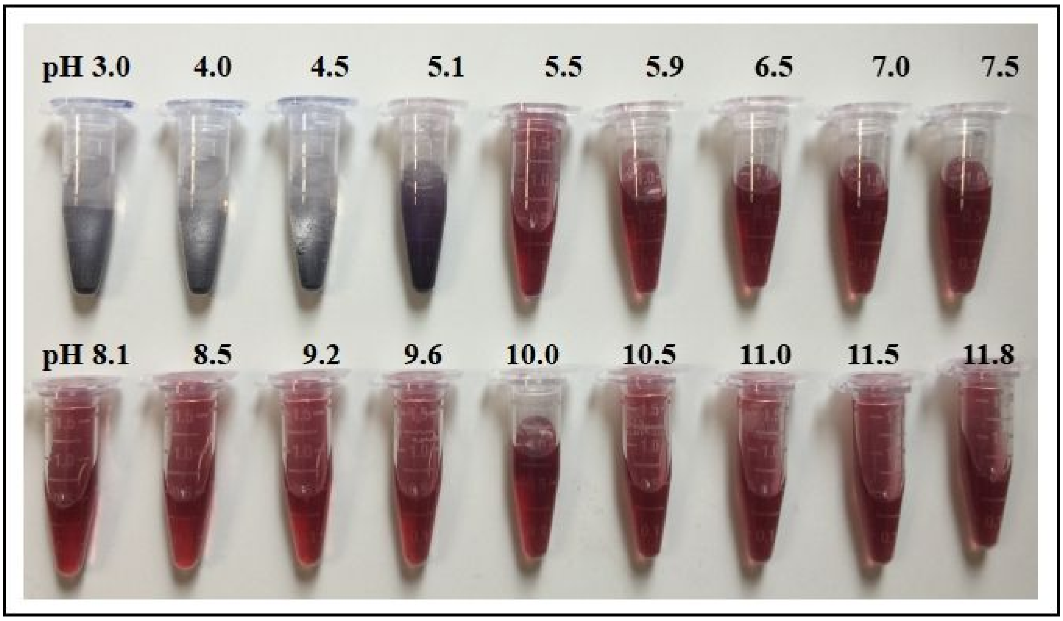

Figure S5. AuNP suspension color change after addition of CRGD-COOH solution.

Optimization of AuNP Conjugation with CRGD Peptides and Their Characterization

When considered the results of AuNP suspension color change after addition of CRGD-NH_2 and CRGD-COOH, whose isoelectric points were 6.1, AuNPs could be conjugated with CRGD-NH_2 at basic conditions, especially after pH 10.0, and with CRGD-COOH at original pH of AuNP suspension. Thus, 1 ml of 10 nM 13 nm AuNPs were conjugated with 20 and 25 µl of 1 mg/ml CRGD-NH_2 at pH 11.0 and pH 11.5 whereas they were functionalized with CRGD-COOH at pH 5.9. The conjugates were shaken for overnight. The white light images of the suspensions of naked AuNPs, AuNP-CRGD-NH_2 and AuNP-CRGD-COOH conjugates were shown in Figure S5. As seen in the photo, there was no AuNP aggregation after conjugation with both CRGD peptides, and so the color of the suspensions was clear and red.

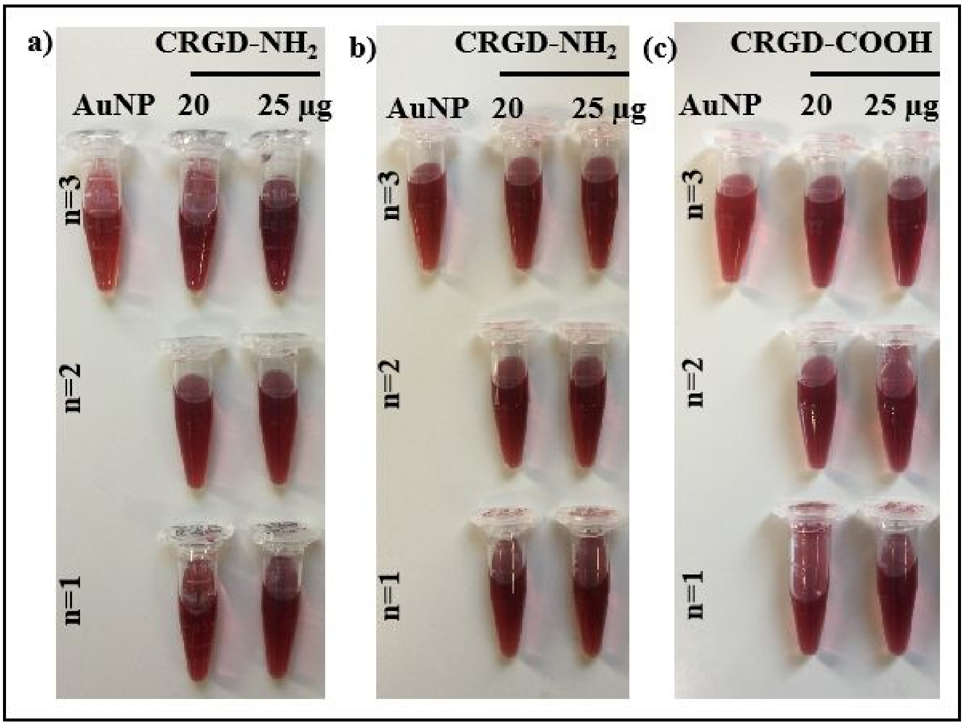

Figure S6. White light images of suspensions of naked AuNPs and AuNPs modified with $CRGD-NH_2$ (a) at pH 11.0 (b) at pH 11.5 and (c) naked AuNPs and AuNPs modified with CRGD-COOH at 5.9. "n" indicates replicate number.

The naked AuNPs, AuNP-CRGD conjugates were characterized by UV/Vis spectroscopy and the comparative UV/Vis spectra of naked AuNPs, $AuNP-CRGD-NH_2$ and AuNP-CRGD-COOH conjugates were given in Figure S6. The SPR of naked AuNPs was at 519 nm. When considered the SPR of $AuNP-CRGD-NH_2$ conjugates, the conjugates at pH 11.0 had 2 nm red-shift in SPR while the conjugates at pH 11.5 had 3 nm red-shift. On the other hand, AuNP-CRGD-COOH conjugates had SPR at 522 nm. The shifts of SPR represented the successful functionalization.

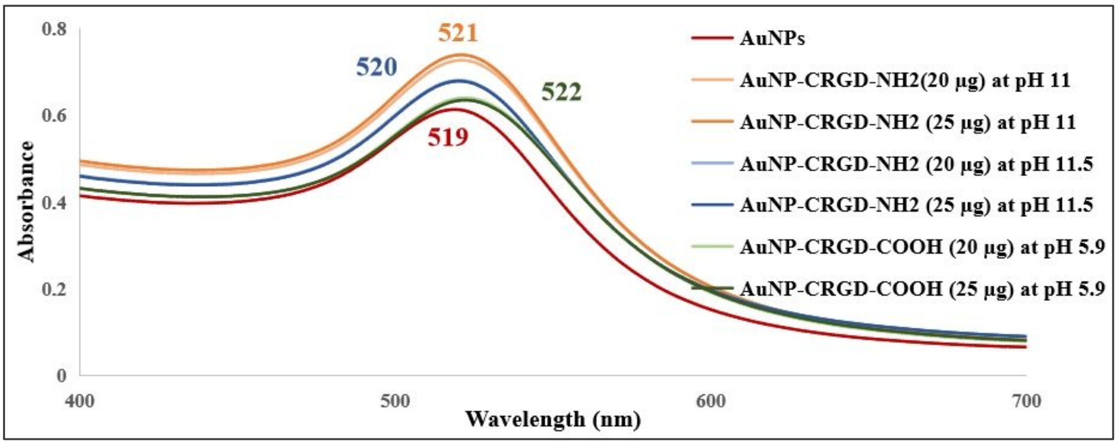

Figure S7. Comparative UV/Vis spectra of naked AuNPs, $AuNP-CRGD-NH_2$ and AuNP-CRGD-COOH conjugates.

The naked AuNPs, AuNP-CRGD conjugates were also characterized by DLS. The hydrodynamic sizes and zeta potentials of the triplicates were measured three times and total collected nine measurements were averaged. The average hydrodynamic size and zeta potentials of naked AuNPs and AuNP-CRGD conjugates were given with standard deviations in Table S1. The average hydrodynamic size of naked AuNPs was 11.0 nM and their average zeta potential was -11.9 mV. After conjugation, it was observed that the sizes were grown, and the surface charges were more negative when compared to naked AuNPs. The sizes of AuNP-CRGD-NH_2 conjugates were 1-2 nm larger and AuNP-CRGD-COOH conjugates were 2 nm larger than naked AuNPs. The zeta potentials of AuNP-CRGD-NH_2 were more negative than AuNP-COOH. The two different pHs, 11.0 and 11.5, and two different concentrations, 20 and 25 μg/ml, for the AuNP-CRGD-NH_2 suspension, were used to test the stability while a pH of 5.9 was used for the AuNP-CRGD-COOH suspension at 20 and 25 μg/ml concentrations.

Table S1. Average hydrodynamic sizes and zeta potentials of naked AuNPs, AuNP-CRGD-NH_2 and AuNP-CRGD-COOH conjugates.

Sample	Size (nm)	Zeta Potential (mV)
AuNPs	11.1 ± 0.4	-11.9 ± 0.3
AuNP-CRGD-NH_2 (20 μg) at pH 11.0	12.1 ± 0.9	-35.9 ± 1.8
AuNP-CRGD-NH_2 (25 μg) at pH 11.0	11.8 ± 0.8	-35.0 ± 3.2
AuNP-CRGD-NH_2 (20 μg) at pH 11.5	12.7 ± 0.9	-32.8 ± 2.9
AuNP-CRGD-NH_2 (25 μg) at pH 11.5	13.4 ± 1.2	-22.4 ± 3.0
AuNP-CRGD-COOH (20 μg) at pH 5.9	13.2 ± 0.8	-17.6 ± 2.1
AuNP-CRGD-COOH (25 μg) at pH 5.9	13.0 ± 0.5	-18.3 ± 3.9

The naked AuNPs and AuNP-CRGD conjugates were also characterized by 1% Agarose gel electrophoresis. The images of the agarose gels after run were seen in Figure S7. In the 1st and 2nd wells of the first gel, AuNP-CRGD-NH_2, conjugated at pH 11.0, were loaded, in the 3rd and 4th wells, AuNP-CRGD-NH_2, conjugated at 11.5, were loaded. According to the result, it was clearly seen that CRGD-NH_2 could cover AuNP surfaces at both pH conditions, but interestingly the bands of the conjugate at pH 11.0 were denser and that of at pH 11.5 run through the gel faster. This showed that the surfaces of AuNPs in the suspension were equally functionalized with CRGD-NH_2 and better in the condition of pH 11.0. Moreover, the concentrations of CRGD-NH_2, 20 and 25 μl of 1 mg/ml, were sufficient to cover the surfaces of whole AuNPs in the suspension. To compare AuNP-CRGD-NH_2 and AuNP-CRGD-COOH conjugates, they were loaded and run in the same gel, as seen in the second gel. In the 5th and 6th wells, AuNP-CRGD-NH_2 at pH 11.5 were loaded and in the 6th and 7th wells, AuNP-CRGD-COOH were loaded. The bands of AuNP-CRGD-NH_2 at pH 11.5 were seen broad whereas the bands of AuNP-CRGD-COOH were denser and run through the gel faster. Moreover, the bands of the same conjugates were parallel to each other, so they coat the AuNP surfaces with less difference. As a result, it can be decided that 20 μl of 1 mg/ml peptides were enough to cover the surfaces of whole AuNPs in the suspension,

AuNPs should be conjugated with CRGD-NH_2 at pH 11.0, and AuNP-CRGD-COOH run through the gel faster than AuNP-CRGD-NH_2.

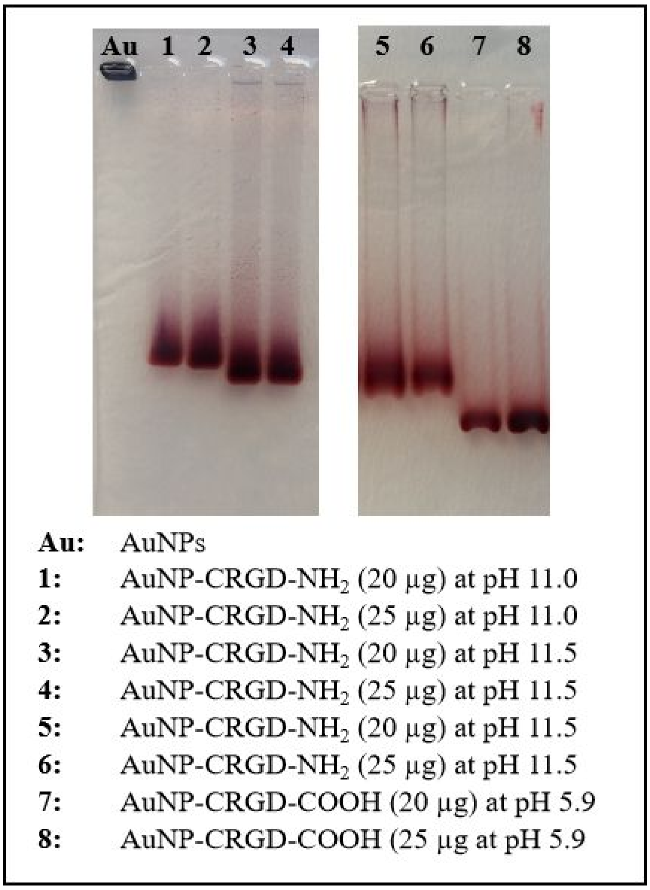

Figure S8. White light image of 1% Agarose gel loaded naked AuNPs and AuNP-CRGD conjugates.

Table S2. Size and surface charge of AuNPs in cell culture media.

	Suspension		Medium Dispersion				
				DMEM+%5 FBS		DMEM/F12+%10 FBS	
	Size (nm)	Zeta potential (mV)	Dispersion concentration (nM)	Size (nm)	Zeta potential (mV)	Size (nm)	Zeta potential (mV)
AuNPs	11,7 ± 0,7	-17,6 ± 0,6	0.1	77,0	-12±0,8	63,9	-18,7±2,1
			0.5	53,7	-9,4±2,3	77,6	-13,9±1,2
			1.0	53,4	-12,1±1	64,3	-12,3±0,9
			2.5	52,0	-16,7±1,6	62,4	-13,6±0,6
AuNP-CRGD-NH_2	18,9 ± 0,8	-35,9 ± 2,7	0.1	122,4	-15,1±0,7	19,8	-12±1,2
			0.5	164,2	-13,7±0,9	28,1	-15,7±1,3
			1.0	333,5	-14,5±2,6	20,3	-17,8±0,9
			2.5	395,3	-17,4±0,7	282,9	-16±0,7
AuNP-CRGD-COOH	12,8 ± 1,2	-9,5 ± 0,3	0.1	175,8	-15,6±1,3	49,9	-6,225±1
			0.5	197,3	-16,4±1,2	116,3	-12,2±0,03
			1.0	166,9	-16,9±1,3	134,4	-13,9±0,9
			2.5	91,3	-17,8±1,2	118,2	-15,7±1,5

Cell Culture

A549 cell line (adenocarcinomic human alveolar basal epithelial cells) was cultured in DMEM/F-12 (with 4500 mg/L) supplemented with 10% FBS, 100 unit/ml Penicilin, 100 µg/ml Streptomycin and 2 mM L-Glutamine, and BEAS-2b cell line (human bronchial epithelial cell line) was cultured in DMEM (with 4500 mg/L) supplemented with 5% FBS, 100 unit/ml Penicilin, 100 µg/ml Streptomycin in an incubator at 37°C with 5% CO_2 humidified atmosphere. When A549 cells reached to 90-95% confluency and BEAS-2b cells reached to 75-80% confluency, they were passaged.

The cells were treated with either naked AuNPs or AuNP-CRGD conjugates with increasing concentrations as 0.1, 0.5, 1.0 and 2.5 nM for 24h. Before their addition to the cell culture media, the AuNP conjugates suspensions were washed with dH_2O once by centrifuging at 13,000 rpm for 20 min. Then, 970 µl of supernatant was discarded and 950 µl of deionized water was added and so all NPs were suspended in total of 1 mL suspension. The cells were incubated with 1 mL of that (5.37×10^{12} NPs) for Apoptosis Necrosis Assay, cell cycle determination and NP uptake studies, and 2 ml of that (10.74×10^{12} NPs) for clonogenic assay.

Cellular Uptake

Cellular uptake of NPs was examined using flow cytometry. A549 cells (50,000), and BEAS-2b cells (42,000) were seeded in each well of 24 well plates and incubated at 37°C in humidified atmosphere under 5% CO_2 for 24h. The cells were exposed to increasing concentrations of AuNPs and their peptide conjugates, and incubated for 24 h in a humidified incubator. After incubation, the cell culture medium in wells were collected into

1.5 mL Eppendorf tubes. The attached cells were incubated with 200 µl of trypsin-EDTA solution for 5 min at 37°C in humidified incubator. In order to finish trypsin activity, 400 µl of collected medium was added in the wells. The detached cells were harvested into same Eppendorf tubes and agitated at 2500 rpm for 5 min. After centrifugation, the cells were suspended in 1x PBS and immediately analyzed by flow cytometry. The quadrant gate on SSC vs FSC plot was used to determine the cellular uptake of NPs. Based on the calculation of quadrant percentages by software, the results were drawn as a clustered column graph and were analyzed with two paired Student's t-test statistically to examine the cellular uptake of cells treated with increasing concentrations of NPs in comparison to negative control cells.

Apoptosis/Necrosis Assay

In order to determine the rate of apoptotic and necrotic cells of the cell population upon NP exposure, Annexin V-FITC apoptosis and necrosis detection kit from Calbiochem (Merck Millipore) was applied according to the manufacturer's instruction. A549 cells (50,000) and BEAS-2b cells (42,000) were seeded into 24 well cell culture plates (n=3) and incubated for 24 h in the incubator at 37°C with 5% humidified atmosphere. After 24 h, the cells were treated with either 10% DMSO as positive control or 0.1, 0.5, 1.0 and 2.5 nM medium dispersed NPs and incubated for 24 h. Upon NP exposure, the cell culture medium containing detached cells in the wells were collected into 1.5 ml Eppendorf tubes. The attached cells were incubated with 200 µl of trypsin for 5 min at incubator. A 400 µl of collected cell culture medium was added into trypsinized cells due to inactivate trypsin. The detached cells were collected in the same tubes including cell culture medium. The samples were centrifuged at 2500 rpm for 5 min at 4°C to obtain cells. Then, the supernatants were aspirated, and they were washed with 1x PBS. They were centrifuged at 2500 rpm for 5 min, again. Based on manufacturer's instruction, 1x binding buffer was prepared from 10x binding buffer with deionized water. 0.5 µl Annexin V-FITC reagent and 1 µl PI reagent per sample was added into 1x binding buffer to prepare the dye mix. After washing with 1x PBS, one negative control was not stained with any dyes to analyze unstained cells, one negative control was stained with only Annexin V-FITC in order to set up green detector voltage, one negative control was stained with only PI due to adjust red detector voltage, and finally the other negative control samples, positive control samples and NP treated samples were stained with both dyes in 200 µl 1x binding mix for the purpose of apoptosis and necrosis detection. All stained cells were incubated with dyes at dark for 15 min. The samples were kept on at 4^0C until analysis. For each sample, 20 000 cells were counted and analyzed by using on Guava easy-Cyte™ 5 (Merck Millipore) benchtop flow cytometer. According to calculation of the quadrants ratio of cell population by software, the results were drawn as a 2-D stacked column graph.

Clonogenic Assay

The colony formation ability of cell was visualized by clonogenic assay. For this assay, 100 cells for A549 and BEAS-2b cells were seeded into each well of 6 well plates (n=3) and waited for cell's attachment for 24 h at 37°C and 5% CO_2 humidified incubator. Then, the

cells were treated with 2 ml either 10% DMSO as positive control or medium dispersed 0.1, 0.5, 1.0 and 2.5 nM NPs. A549 cells were incubated for seven days whereas BEAS-2b cells were for 10 days in the humidified incubator until each seeded single cells formed colonies with at least 50 cells. During 7-10 days incubation, the media on cells were not changed and the plates were not moved anywhere to make able to create a steady condition. In the final days of the incubation, the colonies of negative controls were observed under a light microscope and the cells in each colony were counted. If the number of cells in colonies were close to 50, the incubation was stopped. The media into the wells were removed and the colonies were stained with crystal violet by incubating with dye for 15 min. Then, the dye was taken away and the plates were washed with water until all dye was removed from the plate. The washed plates were left to dry. The violet-colored colonies were counted. The results were drawn a clustered column graph and were analyzed with two paired Student's t-test statistically to investigate the survival ability of cells exposed to increasing concentrations of NPs in comparison to negative control cells by forming colonies.

Cell Cycle Evaluation

The cell cycle progression of A549 and BEAS-2b cells were analyzed for 24h treatment with either naked AuNPs or AuNP-peptide conjugates by flow cytometry. A549 cells (50,000) and BEAS-2b cells (42,000) were seeded in each well of 24 well plates (n=3) and incubated for 24h in an incubator at 37°C with 5% CO_2 humidified atmosphere. Then, the cells were treated with 0.1 µM colchicine as a positive control and 0.1, 0.5, 1.0 and 2.5 nM of medium dispersed NPs for 24 h, and the cell culture media in wells were collected into 1.5 ml Eppendorf tubes. The attached cells were incubated with 200 µl of trypsin-EDTA solution for 5 min at 37^0C in humidified incubator. 400 µl of collected medium was added into detached cells to terminate trypsin activity. The collected cells were centrifuged at 2500 rpm for 5 min at 4^0C. The supernatant was aspirated, and the cells were suspended in 1x PBS. After the centrifugation at the same speed and time, the cells were fixed with 500 µl of 70 % ice-cold ethanol (v/v, ethanol in water) by gently mixing and kept at -20°C at least overnight. The fixed cells were agitated at 2500 rpm for 5 min at 4°C. The supernatant was aspirated, and the cells were suspended in 500 µl of 0.1 % ice-cold Triton X-100 (v/v, Triton X-100 in 1x PBS) and incubated for 20 min at room temperature to make cells permeabilized. After incubation with Triton X-100, the cells were centrifuged and the cells were suspended in 200 µl of 100 µg/ml of RNase solution (v/v, RNase solution in 1x PBS) and incubated for 30 min at 37°C to prevent attachment of propidium iodide (PI) to RNAs, which gave positive wrong results. Finally, PI staining was carried out as the cells were stained with 1 µg/ml for 15 min in dark, except one negative control, which called as unstained. Then, their cell cycle progressions were analyzed on Red Width vs Red Area plot on flow cytometry software. The cell cycle phases as G0/G1, S and G2/M, were adjusted by considering those of negative and positive controls. According to the calculation of cell cycle phase percentages by software, the results were drawn as a 2-D stacked column graph.

References

[1] W. Haiss, N.T.K. Thanh, J. Aveyard, D.G. Fernig, Determination of size and concentration of gold nanoparticles from UV− Vis spectra, Anal. Chem. 79 (2007) 4215–4221.
